# Extracellular Vesicles Profile and Risk of Venous Thromboembolism in Patients with Diffuse Large B-Cell Lymphoma

**DOI:** 10.3390/ijms26125655

**Published:** 2025-06-12

**Authors:** Vladimir Otasevic, Charlotte Gran, Natasa Milic, Jelena Ivanovic, Sofija Kozarac, Vojin Vukovic, Biljana Mihaljevic, Nikolina Dukic, Jelena Vladicic Masic, Jawed Fareed, Jovan Antovic, Darko Antic

**Affiliations:** 1 Lymphoma Center, Clinic for Hematology, University Clinical Center of Serbia, 11000 Belgrade, Serbia; vladimirota@hotmail.com (V.O.); jivanovic09@gmail.com (J.I.); sofijasarac@gmail.com (S.K.); vojinvukovic@yahoo.com (V.V.); bilja.mihaljevic@gmail.com (B.M.); 2 Parexel International, Durham, NC 27713, USA; 3 Department of Molecular Medicine and Surgery, Karolinska Institutet, Department of Clinical Chemistry, Medical Diagnostics Karolinska, Karolinska University Hospital, SE-171 76 Stockholm, Sweden; charlotte.gran@regionstockholm.se (C.G.); jovan.antovic@ki.se (J.A.); 4 Institute for Medical Statistics and Informatics, Faculty of Medicine, University of Belgrade, 11000 Belgrade, Serbia; silly_stat@yahoo.com; 5 Faculty of Medicine, University of Belgrade, 11000 Belgrade, Serbia; 6 Department for Internal Medicine, Faculty of Medicine Foca, University of East Sarajevo, 73300 Foca, Republic of Srpska, Bosnia and Herzegovina; jelenavladicicmasic@gmail.com; 7 Loyola University Medical Center, Maywood, IL 60153, USA; jfareed@luc.edu

**Keywords:** extracellular vesicles, diffuse large B-cell lymphoma, venous thromboembolism, cancer-associated thrombosis

## Abstract

Thrombosis is a common complication in cancer patients, with a substantial impact on morbidity and mortality. Diffuse large B-cell lymphoma (DLBCL) and other aggressive lymphomas carry a high venous thromboembolism (VTE) risk. Extracellular vesicles (EVs) have gained attention in recent research as a new potential biomarker for VTE development. To determine the profile and association of EVs with VTE in patients with DLBCL, we conducted a prospective cohort study on 62 patients diagnosed with DLBCL. A total of 11 patients (17.7%) developed VTE. The concentrations of platelet-derived EVs (PEVs), E-selectin+ EVs, P-selectin+ EVs, tissue factor (TF)-positive/CD20+ EVs, TF−/CD19+ EVs, TF−/CD45+ EVs, and TF−/CD20+ EVs were significantly higher in DLBCL patients compared to healthy controls. In contrast, the concentration of TF− PEVs was significantly lower in DLBCL patients compared to healthy controls. No statistically significant differences were observed in the concentrations of the EV profiles among the DLBCL patients with and without VTE. Using Cox regression analysis, we found that none of the observed EV populations demonstrated an association with overall survival (OS). In conclusion, patients with DLBCL have elevated concentrations of distinct EV populations—in particular, PEVs, E-selectin EVs, P-selectin EVs, TF+/CD20+ EVs, and TF− DLBCL/B-cell EVs (CD19, CD20, CD45)—compared to healthy controls. DLBCL patients exhibit a specific EV profile, which is not significantly related to the risk of VTE and OS outcomes. Our data provide an intriguing insight into EV profiles in patients with DLBCL. Additional research is needed to elucidate these findings further.

## 1. Introduction

Lymphomas represent a heterogeneous group of malignant clonal neoplasms of lymphoid cells. Thrombosis represents a common complication in cancer patients and impacts the clinical course of the disease, as well as the mortality and morbidity in cancer patients. Cancer-associated thrombosis (CAT) is a leading cause of death in cancer patients receiving chemotherapy [1]. Lymphomas, especially aggressive ones, such as diffuse large B-cell lymphoma (DLBCL), carry an increased risk of venous thromboembolism (VTE) [2]. In addition to the well-established risk factors for VTE, extracellular vesicles (EVs) have gained attention in recent research as a possible new biomarker for VTE. In its latest update of guidelines, the International Society for Extracellular Vesicles (ISEV) endorses EVs (also known as microparticles, microvesicles, ectosomes, or exosomes) as a generic term for lipid bilayer particles that cannot replicate (do not contain a functional nucleus), and which are released from the cell [3]. Extensive research over the past two decades has been crucial for a paradigm shift toward understanding the sophisticated role of EVs in numerous physiological and pathological processes. EVs are released by different cells: platelets, erythrocytes, lymphocytes, dendritic cells, endothelial cells, and tumor cells [4,5]. The size of EVs ranges from 30 to 100 nm, and their content varies. They are a crucial mediator in the intercellular communication between cells, along with the secretion of small soluble molecules and direct cell–cell contact [6]. EVs have been shown to play substantial roles in promoting and suppressing cell death and inflammation regulation in physiological and pathological processes [7]. The number of EVs in cancer patients’ blood is twice as high as that in normal human blood [8]. Cancer cells conduct their transfer to recipient cells through EVs. Materials that can be passed on include proteins with pro-tumorigenic roles, messenger ribonucleic acid (mRNA), and micro-ribonucleic acid (miRNA), which influence the cellular program of target cells both at the transcriptional and post-transcriptional levels. Additionally, EVs can have an important diagnostic role in non-invasive cancer detection through liquid biopsies, where cancer EVs—such as specific miRNAs or mRNAs—are quantified in blood, urine, or other body fluids. The role of EVs in CAT relies on the negatively charged plasma membrane of EVs, which serves as a catalytic surface for the vitamin K-dependent clotting factors FII, FVII, FIX, and FX. Phosphatidylserine (PS) on EVs binds clotting factors and increases the catalytic efficiency of phospholipid-dependent coagulation reactions, such as the activation of tenase and prothrombinase complexes [9]. EVs also display procoagulant activity, which depends on their mechanism of formation and cargo content, including tissue factor (TF), inflammatory molecules, podoplanin, P-selectin, and P-selectin glycoprotein ligand-1 (PSGL-1) [9,10,11].

The processes and mechanisms of inflammation development and CAT in cancer patients are complex and partially overlapping. Therefore, this study aims to examine the association of EVs and VTE in patients with DLBCL and to characterize the EV profiles in these patients.

## 2. Results

This study included 62 patients with a median age of 59 years (range 20–87 years); 51.6% were males and 48.4% were females. Most of the patients (72.6%) had a good performance status (ECOG PS ≤ 1, Eastern Cooperative Oncology Group performance status). B symptoms appeared in 29 (46.8%) patients, bulky tumor masses (defined as lymphoma masses or conglomerates of lymph node masses that measure ≥ 7 cm) in 22 (35.5%) patients, and disease relapse in 3 (4.8%) patients. Most patients included in the study had at least one extranodal disease localization (71%), while mediastinal involvement was recorded in 27.4% of DLBCL patients. Central nervous system (CNS) involvement was observed in five DLBCL patients (8.1%). Patients were followed prospectively for 32 months. The rate of VTE was 17.7% (11 patients): 45.5% before the initiation of specific hematological therapy, 36.4% during treatment, and 18.1% after the completion of specific hematological treatment. The frequency of VTE according to localization was as follows: three patients had deep vein thrombosis (DVT), one had portal vein thrombosis, one had subclavian vein thrombosis, one had mesenteric venous thrombosis, one had superficial thrombophlebitis, one had internal jugular vein thrombosis, and four patients were diagnosed with pulmonary embolism (PE). One patient had VTE simultaneously in two sites. There were no deaths caused by VTE in our cohort of patients. The use of thromboprophylaxis in patients with and without VTE was not shown to have a statistically significant impact (18.2% vs. 21.6%, *p* = 0.802, respectively). The demographic and clinical characteristics of the patients, depending on the presence of VTE, are shown in Table 1. Patients with both DLBCL and VTE had a significantly higher frequency of disease relapse (18.2% vs. 2% of patients, *p* = 0.023) and bulky disease (63.6% vs. 29.4% of patients, *p* = 0.031). The characterization of EVs in patients with DLBCL is shown in Table 2. The concentrations of EVs in patients with DLBCL were compared with those in healthy controls. Additionally, the different profiles of these two populations were compared (Annexin V for PS; CD42b/CD61 for PEVs; CD142 for TF; CD19, CD20, and CD45 for DLBCL/B-cell population; and CD62P/CD62E for P-selectin/E-selectin). The concentrations of PEVs, E-selectin+ EVs, P-selectin+ EVs, TF+/CD20+ EVs, TF−/CD19+ EVs, TF−/CD45+ EVs, and TF−/CD20+ EVs were significantly higher in DLBCL patients compared to healthy controls (*p* = 0.018, *p* = 0.008, *p* = 0.042, *p* < 0.001, *p* < 0.001, *p* < 0.001, *p* < 0.001, respectively). Conversely, the concentration of TF− PEVs was higher in the control group than in the DLBCL patient group (*p* = 0.041). However, there were no statistically significant differences in the concentrations of the following EVs between the two groups: Annexin V+ (*p* = 0.326), TF+ (*p* = 0.750), CD19+ (*p* = 0.437), CD45+ (*p* = 0.350), CD20+ (*p* = 0.066), TF+ PEVs (*p* = 0.658), TF+/CD19+ (*p* = 0.490), TF+/CD45+ (*p* = 0.996), TF+/CD19+/CD20+ (*p* = 0.290), and TF−/CD19+/CD20+ (*p* = 0.690).

No significant difference was observed in the profile of total Annexin V+ EV concentrations between DLBCL patients with VTE and those without VTE (*p* = 0.513). Also, no significant differences were found in the concentrations of other EVs included in the research (PEVs, TF+, E-selectin+, P-selectin+, CD19+, CD45+, CD20+, TF+ PEVs, TF+/CD19+, TF+/CD45+, TF+/CD20+, TF+/CD19+/CD20+, TF− PEVs, TF−/CD19+, TF−/CD45+, TF−/CD20+, TF−/CD19+/CD20+) with regard to VTE development (Table 3).

Using Cox regression analysis, after a median follow-up of 32 months, it was found that the total concentration of Annexin+ EVs did not show an association with overall survival (OS) (*p* = 0.858). Additionally, with respect to OS, no statistically significant differences were observed in the concentrations of other EVs (PEVs, TF+, E-selectin+, P-selectin+, CD19+, CD45+, CD20+, TF+ PEVs, TF+/CD19+, TF+/CD45+, TF+/CD20+, TF+/CD19+/CD20+, TF− PEVs, TF−/CD19+, TF−/CD45+, TF−/CD20+, TF−/CD19+/CD20+), as shown in Table 4.

## 3. Discussion

VTE is one of the leading causes of death in cancer patients who receive chemotherapy, including patients with lymphoma, especially those with DLBCL and other aggressive lymphomas [1]. This study examined the EV profile in patients with DLBCL and its relationship with VTE occurrence. In a prospective cohort of DLBCL patients, the concentrations of PEVs, including E-selectin+ EVs, P-selectin+ EVs, TF+/CD20+ EVs, TF−/CD19+ EVs, TF−/CD45+ EVs, and TF−/CD20+ EVs, were significantly higher compared to those in healthy controls. In contrast, the concentration of TF− PEVs was significantly lower in DLBCL patients compared to that in healthy controls. Regarding VTE occurrence and OS outcomes, no statistically significant differences were observed in the concentrations of the EVs mentioned above between the DLBCL patient groups.

The average age of the DLBCL patients included in our study was 59 years (range 20–87 years), and 51.6% were males. Most of the patients had ECOG PS 0–1, while disease relapse occurred in three (4.8%) patients. In another study that included patients with DLBCL and follicular lymphoma, the average age was 63 (range 16–92), with 81.5% of patients having an ECOG PS of 0–1 [12]. The rate of VTE occurrence in our patients was 17.7% (11 patients)—45.5% before the initiation of the specific hematological therapy, 36.4% during treatment, and 18.1% after the completion of the specific hematological therapy. Additionally, in our study, patients with DLBCL with VTE had a significantly higher frequency of disease relapse (18.2% vs. 2% of patients, *p* = 0.023). In a previous retrospective cohort study that included patients with various types of lymphoma, the incidence of VTE was 9.8% [13]. In a meta-analysis [14] that included 18,018 lymphoma patients, the incidence of VTE was 6.4%, with a statistically significantly higher incidence in patients with non-Hodgkin lymphoma (NHL) compared to those with Hodgkin lymphoma (HL) (6.5% vs. 4.7%, respectively). In two studies [15,16] focusing exclusively on DLBCL patients, VTE was observed in 11% and 11.1% of patients, respectively. Complementary to our results, the predominant time of VTE occurrence in lymphoma patients was before or within three months of the initiation of hematological treatment [2,16,17,18,19,20,21]. The observed differences in VTE incidence among patients with lymphomas are notable. However, the results of our study are consistent with the results of studies that focused on patients with aggressive lymphomas. Patients with relapsed/refractory lymphoma are at an increased risk of developing VTE [22,23], which aligns with our findings. The clinical course of aggressive lymphomas is associated with a higher likelihood of refractory/relapsed disease, which translates into a greater risk of complications, including VTE.

Our cohort of patients with DLBCL and VTE had a significantly higher frequency of bulky disease (63.6% vs. 29.4% of patients, *p* = 0.031). In univariate analysis, bulky disease, mediastinal involvement, and ECOG PS were identified as prognostic factors for VTE development in patients with lymphoma [13]. Immobilization, a known risk factor for VTE, is often an essential characteristic of patients with PCNSL, in whom the incidence of VTE ranges from 8.4% to 59.5% [2,24,25,26,27]. Nonetheless, none of our patients with PCNSL developed VTE, which could be attributed to the adequate use of thromboprophylaxis (in four out of four patients).

EVs represent a dynamic field of scientific research, with notable heterogeneity related to the methodologies used for EV analysis. All the following techniques are used to analyze EVs: flow cytometry, Western blot, next-generation sequencing, nanoparticle tracking analysis, and transmission electron microscopy [28,29,30]. Each method has its advantages and limitations, ranging from differences in availability, price, and complexity to the interpretation of results. In addition, functional assays are applied to characterize EVs, such as those assessing coagulation (clotting time, thrombin generation), fibrinolysis (plasmin generation), and cellular functions [31,32,33,34]. Concentrations of EVs are increased twofold in the blood circulation of cancer patients compared to healthy controls [8]. The reason for this is still unclear, but it is known that the concentrations of many proteins and lipids that form EVs are elevated in various neoplasms [28,35]. Even though we observed significant differences in several EV populations, the overall concentration of Annexin V+ EVs between DLBCL patients and healthy controls was not significantly different (*p* = 0.326). Another study showed differences in the EV proteome between DLBCL patients and healthy controls in functional, qualitative, and quantitative analyses. In DLBCL patients, CD9+ and CD81+ EVs were found in significantly higher concentrations than in healthy controls [36]. Rutherford et al. [37] investigated the characterization of EVs in patients with DLBCL and found that DLBCL-derived EVs were CD20-positive. They also discovered that DLBCL-derived EVs can be traced to provide insight into the cell of origin. Even though we observed a numerical difference in Annexin V+ EV concentration, it did not reach statistical significance. One possible explanation for this finding could be that DLBCL patients display a higher concentration of specific EVs accompanied by a lower concentration of other EVs. This imbalance could explain the insignificant difference in the total concentration of EVs between DLBCL patients and healthy controls, as observed in our study.

PEVs are released into the circulation by platelets during activation. The concentrations of PEVs in the blood of healthy individuals are estimated to be between 20 EV/μL and 10^9^ EV/μL [36,38]. According to the results of our study, the concentration of PEVs was significantly higher in patients with DLBCL than in healthy controls (*p* = 0.018). However, the concentration of TF− PEVs was higher in the control group than in the DLBCL group (*p* = 0.041). At the same time, no significant difference was found in the concentration of PEVs in DLBCL patients regarding VTE occurrence and OS. To the best of our knowledge, the role of PEVs has not been previously investigated in a homogeneous group of DLBCL patients. Increased concentrations of PEVs have been reported in various solid malignancies (breast, colorectal, gastric, lung, or oral squamous cell carcinoma) [39,40,41,42,43,44]. In a study [45] that involved patients with various malignancies (including 53 patients with lymphoma), a significantly higher concentration of PEVs was found in patients who developed CAT than in those without CAT. Two primary mechanisms have been proposed to account for the prothrombotic activity of PEVs. The first is associated with TF [46]. The second mechanism originates from the exposure of negatively charged phospholipids, which enables the generation of complex bonds with Ca^2+^ ions. These bonds, in turn, bind vitamin K-dependent coagulation factors. In this way, coagulation cascade components are concentrated on the surface of negatively charged membranes, resulting in a drastic increase in the proteolytic conversion rate and the activation of coagulation factors.

Significantly higher concentrations of E-selectin+ EVs, P-selectin+ EVs, TF+/CD20+ EVs, TF−DLBCL/B-cell EVs (CD19, CD20, CD45), and TF−/CD20+ EVs were found in the DLBCL patients in comparison to healthy controls. Conversely, no statistically significant differences in the concentrations of all investigated EV populations were observed among the DLBCL patients regarding VTE development. Next, the concentrations of the EVs mentioned above were not associated with the OS of DLBCL patients. There are very few publications on P-selectin+ EVs and E-selectin+ EVs in patients with malignancies. To our knowledge, no papers specifically address P-selectin+ EVs and E-selectin+ EVs in patients with DLBCL. In contrast, there are more scientific data and evidence on EVs expressing PSGL-1. Thomas et al. [47] demonstrated that pancreatic and lung cancer cells produce EVs expressing TF and PSGL-1 in both human and animal models in vitro. Another study [48] involving patients with unprovoked VTE (without a diagnosis of malignancy) showed an association between PSGL-1+ EVs and the risk of developing unprovoked VTE. Further investigation is required to clarify the role of E-selectin+ and P-selectin+ EVs in DLBCL.

TF expressed by EVs is thought to contribute to TF procoagulant activity in patients with cancer, since TF is an essential component in the relationship between coagulation and cancer. However, it is also possible that other EVs in these patients may induce TF expression in cells, leading to prothrombotic activity [38,49]. In our study, the concentrations of TF+/CD20+ EVs and TF− DLBCL/B-cell EVs (CD19, CD20, CD45) were significantly higher in DLBCL patients than in healthy controls. In a study by Van Es et al. [50], which included patients with advanced stages of different types of malignancies, the thrombin generation test confirmed that high EV-TF procoagulant activity was associated with an increased risk of developing VTE. The strongest association was observed in patients with pancreatic cancer, who also had poorer survival rates [51]. In a review article [52], TF+ EVs (i.e., microparticles) were associated with an increased risk of VTE occurrence in several different malignancies. However, the effects of TF expression in patients with lymphoid malignancies have not been fully elucidated by immunohistochemistry. Recent studies on TF expression in lymphomas have produced inconclusive results: some studies refute the notion that TF originates from malignant cells, while others confirm TF expression in DLBCL [53,54]. Our ambiguous results regarding TF expression in DLBCL patients compared to healthy controls suggest that TF may have limited expression in DLBCL-derived EVs, thereby questioning its role in VTE development in DLBCL patients. This conclusion is supported by the lack of a significant difference in TF+ EV concentrations between DLBCL patients with and without VTE, which indicates a limited impact of TF on VTE development in DLBCL, unlike in many solid tumors. This further emphasizes the complexity and heterogeneity of CAT across different types of malignancies, warranting a disease-specific approach.

Our study has several limitations, primarily the small sample sizes of both the patient cohort and the control group, as well as the limited follow-up period for DLBCL patients. Additionally, the statistical analysis did not account for the impact of confounding variables. These limitations could have impacted the strength of our statistical analyses, potentially affecting the significance of the EV profile in relation to VTE development and OS outcomes in DLBCL patients.

## 4. Methods

A prospective cohort study was conducted in collaboration between the Clinic for Hematology, University Clinical Center of Serbia, and Karolinska University Hospital, Coagulation and Clinical Chemistry Service, Department of Molecular Medicine and Surgery, Karolinska Institute and Clinical Chemistry, Karolinska Medical Diagnostics. The study was approved by the Ethics Committee of the University Clinical Center of Serbia (no. 630/19A). It included 62 patients diagnosed with DLBCL recruited for the research between May 2018 and July 2019. Blood samples for all patients involved in the study were collected before the initiation of specific lymphoma treatment. Comprehensive data pertaining to the characteristics of the patients can be found in the Supplementary Materials. Patients in this study adhered to the standard-of-care approach for assessing venous thromboembolism. Based on laboratory and radiology findings, a diagnosis of VTE was made in the recruited patients. To compare EVs in DLBCL patients, a control group of twenty random healthy volunteers was formed. The control group consisted of pseudonymized healthy volunteers aged 38–64 years, with the majority being female. Participants refrained from using NSAIDs, ASA, or other platelet-affecting medications within 10 days prior to sampling. Their peripheral blood samples were collected at the Karolinska Institute, Stockholm, Sweden. Sampling and sample handling for the control group were performed identically to the patient group.

Venous blood was collected into 4.5 mL 0.109 M citrated vacutainer tubes (Becton Dickinson (BD), Franklin Lakes, NJ, USA) with a draw volume of 2.7 mL per tube. To minimize contamination, a discard tube was collected first and discarded before sampling. Two tubes were collected per test subject for analysis. Within 4 h of collection, samples were centrifuged at 3000× *g* for 10 min at 15 °C. The supernatant (platelet-poor plasma) was carefully removed and aliquoted into Eppendorf plastic tubes (0.4–0.5 mL per aliquot). Plasma aliquots were stored at −80 °C until analysis. Prior to analysis, aliquots were thawed in a 37 °C water bath for five minutes; no freeze–thaw cycles were performed.

The concentration of total EVs and their characterization were determined according to the protocol developed by the Karolinska Institute, Stockholm, Sweden [55]. Briefly, platelet-poor plasma (PPP) aliquots were gently resuspended by pipetting after thawing. For each sample, three FACS tubes were prepared: two staining panels (Tube A and Tube B) and one negative control. To each tube, 20 µL of PPP was added, and the EV-PPP samples were diluted fourfold by adding 60 µL PBS to 20 µL PPP. The negative control contained 20 µL PBS and 20 µL PPP without any antibodies. In Tube A, the staining panel included 10 µL FITC-conjugated Annexin V, 10 µL PE-conjugated CD42b, 5 µL APC-conjugated CD142, 5 µL APC-Alexa750-conjugated CD19, 2 µL BB700-conjugated P-selectin, and 2 µL PE-Viol770-conjugated E-selectin, along with 20 µL PBS with a final concentration of 2.5 mM CaCl_2_. Tube B was stained with 10 µL FITC-conjugated Annexin V, 10 µL PE-conjugated CD42b, 5 µL APC-conjugated CD142, 5 µL APC-Alexa750-conjugated CD19, 5 µL Starbright-blue700-conjugated CD20, and 2 µL PE-Viol770-conjugated CD45, along with 20 µL PBS with CaCl_2_ concentration as above.

After the addition of staining reagents, all tubes were briefly vortexed and incubated for 30 min at room temperature in the dark. The incubation was ended by adding 500 µL PBS to each tube to prepare the samples for flow cytometry analysis. Megamix-Plus SSC (Diagnostica Stago, Düsseldorf, Germany) was used to measure the size of EVs. All samples were analyzed on a BD FACS Canto flow cytometer. EVs were defined as vesicles of size ≤1.0 µm that were positive for Annexin V. Flow cytometry data analysis was performed using FlowJo software (version 10.8.1, BD, Ashland, OR, USA). Plots from the negative control, providing an example of the gating strategy, are included in Supplementary Figure S1. The concentration of EVs was calculated using the following formula: Concentration (µL) = (Counts × sample volume in a test tube)/(volume of samples analyzed in 90 s × volume of plasma sample) [55]. The following panel of markers was used: Annexin V for PS; CD42b/CD61 for platelet-derived extracellular vesicles (PEVs); CD142 for TF; CD19, CD20, and CD45 for DLBCL/B-cell population; and CD62P/CD62E for P-selectin or E-selectin. A specific gating algorithm was used to identify EVs. Samples were stained with Annexin V-FITC (fluorescein isothiocyanate).

### Statistical Analysis

Numerical data were presented as medians with ranges or 25–75th percentiles. Categorical variables were summarized as absolute numbers with percentages. The normality of the data distribution was assessed using the Kolmogorov–Smirnov test. Differences between DLBCL patients and healthy controls, as well as between patients with lymphoma who developed thrombosis and those who did not, were assessed using the Mann–Whitney test for numerical variables and the chi-square test for categorical variables. Cox proportional regression was used to assess the survival of DLBCL patients. Statistical analysis was performed using IBM SPSS statistical software (SPSS for Windows, release 25.0, SPSS, Chicago, IL, USA) with a statistical significance level of *p* < 0.05.

## 5. Conclusions

In conclusion, patients with DLBCL have increased concentrations of distinct EV populations, particularly PEVs, E-selectin EVs, P-selectin EVs, TF+/CD20+ EVs, and TF− DLBCL/B-cell EVs (CD19, CD20, CD45), compared to healthy controls. Although DLBCL patients exhibit a specific EV profile, this profile does not significantly correlate with the risk of VTE and/or OS outcomes. Our data provide intriguing insights into the EV profiles of patients with DLBCL. Further research, possibly with a larger cohort of DLBCL patients, is needed to better elucidate these findings.

## Figures and Tables

**Table 1:** Demographic and clinical characteristics of DLBCL patients.

Demographic/Clinical Characteristic	DLBCL pts with VTE	DLBCL pts Without VTE	*p*
Age, median (range)	64 (22–74)	56 (20–87)	0.568
Male/female	4/7	28/23	0.264
Relapse, *n* (%)	2 (18.2)	1 (2)	0.023
B symptoms, *n* (%)	6 (54.5)	23 (45.1)	0.569
Bulky disease, *n* (%)	7 (63.6)	15 (29.4)	0.031
Extranodal localization, *n* (%)	6 (54.5)	38 (74.5)	0.186
Mediastinal involvement	4 (36.4)	13 (25.5)	0.463
CNS involvement, *n* (%)	0	5 (8.1)	0.279
PCNSL DLBCL	0	4 (6.4)	
Dissemination of the disease to CNS	0	1 (1.6)	
Hemoglobin level, g/L, median (range)	119 (96–149)	129 (51–166)	0.257
White blood cell count, ×10^9^/L, median (range)	5.8 (3.8–13.7)	6.9 (2.2–20.6)	0.063
Platelet count, ×10^9^/L, median (range)	295 (103–467)	265 (29–570)	0.775
ECOG PS > 1, %	5 (45.5)	12 (23.5)	0.139

DLBCL—diffuse large B-cell lymphoma; pts—patients; CNS—central nervous system; PCNSL—primary CNS lymphoma; ECOG PS—Eastern Cooperative Oncology Group performance status.

**Table 2:** Characterization of EVs in patients with DLBCL and healthy controls.

EVs	DLBCL pts, All		Healthy Controls		*p*
	Median ×10^9^ EV/L	IQR ×10^9^ EV/L	Median ×10^9^ EV/L	IQR ×10^9^ EV/L	
Annexin V+	423	155–828	407	126–615	0.326
PEVs	228	89–449	158	69–207	0.018
TF+	140	50–272	174	56–221	0.750
E-selectin+	151	74–325	90	47–133	0.008
P-selectin+	306	138–628	252	88–292	0.042
CD19+	7	3–10	6	4–8	0.437
CD45+	137	71–276	158	32–212	0.350
CD20+	272	109–587	229	56–340	0.066
TF+ PEVs	280	104–539	313	97–419	0.658
TF+/CD19+	72	26–129	105	32–152	0.490
TF+/CD45+	295	89–547	339	69–573	0.996
TF+/CD20+	86	51–186	23	7–40	<0.001
TF+/CD19+/CD20+	54	18–109	85	17–166	0.290
TF− PEVs	81	49–184	313	47–419	0.041
TF−/CD19+	30	14–54	10	5–21	<0.001
TF−/CD45+	76	45–156	19	6–32	<0.001
TF−/CD20+	86	51–186	23	7–40	<0.001
TF−/CD19+/CD20+	64	26–129	90	18–173	0.690

EVs—extracellular vesicles; DLBCL—diffuse large B-cell lymphoma; pts—patients; IQR—interquartile range; TF—tissue factor; PEVs—platelet-derived extracellular vesicles; TF—tissue factor.

**Table 3:** Characterization of EVs in DLBCL patients with and without VTE.

EVs	DLBCL pts with VTE		DLBCL pts Without VTE		*p*
	Median ×10^9^ EV/L	IQR ×10^9^ EV/L	Median ×10^9^ EV/L	IQR ×10^9^ EV/L	
Annexin V+	510	152–1213	423	155–789	0.513
PEVs	285	80–659	222	89–434	0.599
TF+	214	32–385	140	50–255	0.747
E-selectin+	205	67–425	148	79–297	0.665
P-selectin+	415	118–861	305	141–590	0.726
CD19+	8	2–9	7	3–10	0.537
CD45+	192	48–347	130	71–273	0.861
CD20+	389	91–817	267	112–541	0.712
TF+ PEVs	356	87–770	269	104–534	0.562
TF+/CD19+	106	17–150	71	32–124	0.993
TF+/CD45+	389	89–804	292	89–544	0.501
TF+/CD20+	91	37–236	80	51–164	0.574
TF+/CD19+/CD20+	82	13–145	52	21–102	0.832
TF− PEVs	81	39–256	82	49–162	0.490
TF−/CD19+	44	9–58	29	14–52	0.692
TF−/CD45+	82	34–204	73	45–145	0.537
TF−/CD20+	91	37–236	80	51–164	0.574
TF−/CD19+/CD20+	121	20–179	61	27–128	0.890

EVs—extracellular vesicles, DLBCL—diffuse large B-cell lymphoma, IQR—interquartile range, TF—tissue factor, PEVs—platelet-derived extracellular vesicles, TF—tissue factor.

**Table 4:** Characterization of EVs in patients with DLBCL regarding the outcome of OS.

EVs	DLBCL pts, Dead		DLBCL pts, Alive		*p*
	Median ×10^9^ EV/L	IQR ×10^9^ EV/L	Median ×10^9^ EV/L	IQR ×10^9^ EV/L	
Annexin V+	446	154–985	404	170–809	0.858
PEVs	261	68–551	211	102–434	0.658
TF+	159	44–332	136	55–253	0.620
E-selectin+	158	82–411	139	73–291	0.525
P-selectin+	343	132–711	273	139–576	0.577
CD19+	9	3–16	7	2–10	0.232
CD45+	170	71–304	124	62–260	0.556
CD20+	330	108–666	243	110–529	0.540
TF+ PEVs	306	87–629	250	119–535	0.716
TF+/CD19+	78	32–172	64	25–124	0.535
TF+/CD45+	308	81–598	241	99–544	0.710
TF+/CD20+	101	55–224	86	49–159	0.609
TF+/CD19+/CD20+	72	22–139	50	17–106	0.577
TF− PEVs	88	50–199	76	49–169	0.804
TF−/CD19+	43	17–56	28	14–50	0.402
TF−/CD45+	90	49–202	76	41–141	0.609
TF−/CD20+	101	55–224	86	49–159	0.609
TF−/CD19+/CD20+	94	27–163	60	24–125	0.466

EVs—extracellular vesicles; DLBCL—diffuse large B-cell lymphoma; pts—patients; IQR—interquartile range; TF—tissue factor; PEVs—platelet-derived extracellular vesicles; TF—tissue factor.

## Data Availability

The datasets used and analyzed during the current study are available from the corresponding author upon reasonable request.

## Supplementary Materials

The following supporting information can be downloaded at https://www.mdpi.com/article/10.3390/ijms26125655/s1.
