# Supplementary material for: Extracellular Vesicles Profile and Risk of Venous Thromboembolism in Patients with Diffuse Large B-Cell Lymphoma
Source: Int J Mol Sci. 2025 Jun 12;26(12):5655. doi: 10.3390/ijms26125655 (PMC12193381; doi:10.3390/ijms26125655)
Supplement: Supplementary file 1 [file ijms-26-05655-s001.zip › Supplementray Figure S1.pdf]

Supplemental Figure S1. Flow cytometry gating strategy for extracellular vesicle (EV) analysis. **(a)** Initial gating using Megamix-Plus SSC beads to identify events corresponding to particles ≤1.0 μm, **(b)** Gating of Annexin V-positive (FITC) EVs, **(c)**-(i) Red boxes indicate gating for populations present in the manuscript. Each combination is detailed below: **(c)** double-positive CD42b and Annexin V, **(d)** double-positive CD142 and Annexin V**, (e)** double-positive CD19 and Annexin V**, (f)** double-positive E-selectin (Panel A) and Annexin V or CD45 (Panel B) and Annexin V**, (g)** double-positive P-selectin (Panel A) and Annexin V or CD20 (Panel B) and Annexin V**, (h)** double-positive EV populations identified as CD19 and CD20 and CD142 expression in the CD19/CD20 double-positive population, **(i)** annexin V-positive EVs further gated to identify CD142-positive EVs co-expressing additional markers, CD42b, CD20, CD19 or CD45, **(j)** healthy control stained with panel A, **(k)** healthy control stained with panel B. All axes are displayed on a logarithmic scale Created in https://BioRender.com

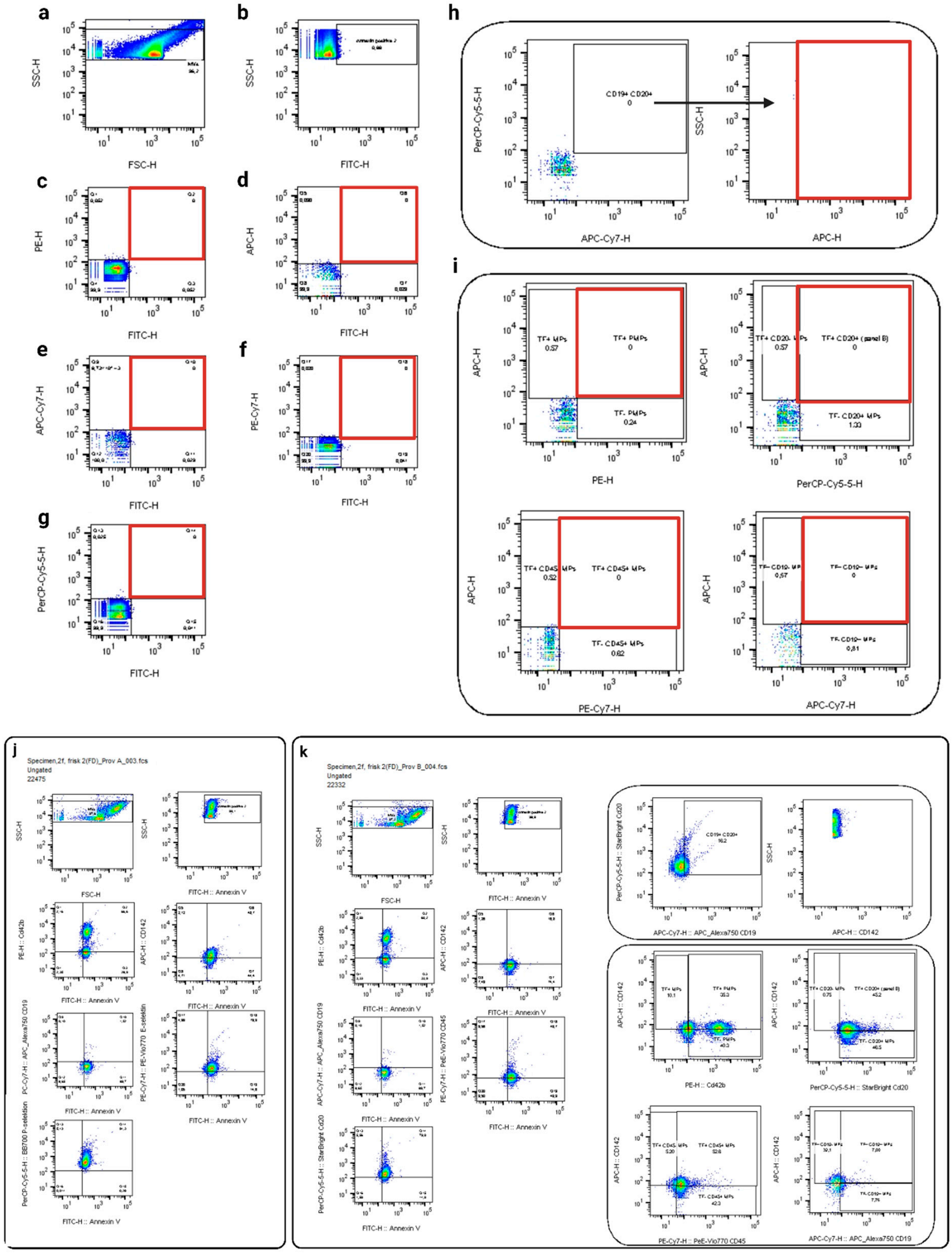

a
b
c
d
e
f
g
h
i
j
k
SSC-H
FSC-H
FITC-H
PE-H
APC-H
APC-Cy7-H
PE-Cy7-H
PerCP-Cy5-5-H
CD19+ CD20+
TF+ MPs
TF+ PMPs
TF- PMPs
TF+ CD20- MPs
TF+ CD20+ (panel B)
TF- CD20+ MPs
TF+ CD45- MPs
TF+ CD45+ MPs
TF- CD45+ MPs
TF- CD19- MPs
TF- CD19+ MPs
Specimen,2f, frisk 2(FD)_Prov A_003.fcs
Ungated
22475
Specimen,2f, frisk 2(FD)_Prov B_004.fcs
Ungated
22332
FITC-H :: Annexin V
PE-H :: Cd42b
APC-H :: CD142
APC-Cy7-H :: APC_Alexa750 CD19
PE-Cy7-H :: PE-Vio770 E-selektin
PerCP-Cy5-5-H :: BB700 P-selektion
PE-Cy7-H :: PeE-Vio770 CD45
PerCP-Cy5-5-H :: StarBright Cd20
