# Supplementary figures and images for: Extracellular Vesicles Profile and Risk of Venous Thromboembolism in Patients with Diffuse Large B-Cell Lymphoma
Source: Int J Mol Sci. 2025 Jun 12;26(12):5655. doi: 10.3390/ijms26125655 (PMC12193381; doi:10.3390/ijms26125655)

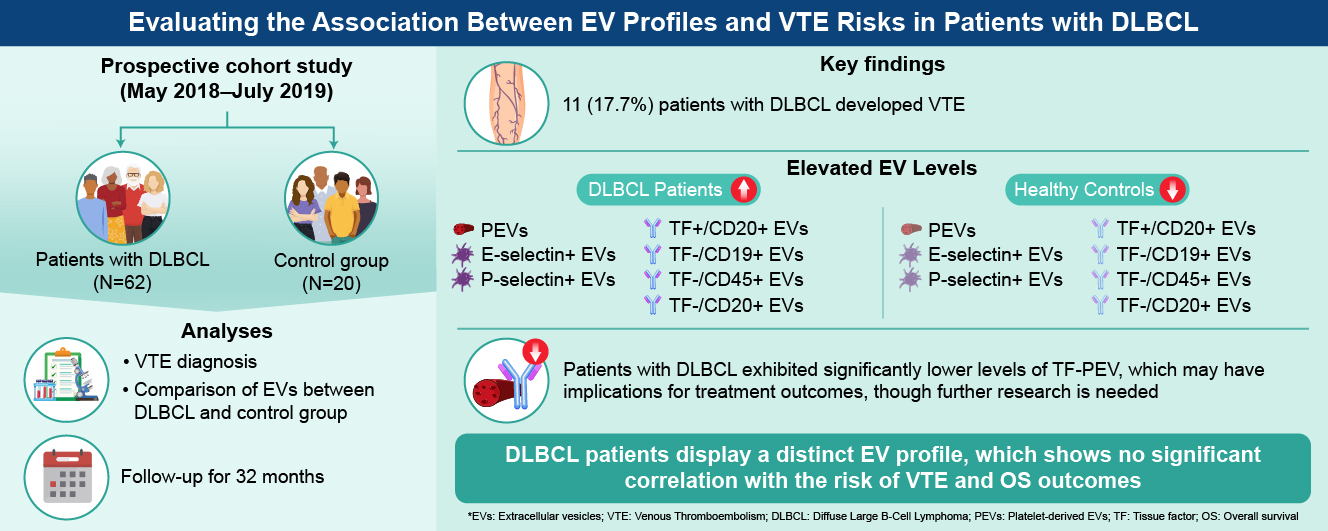

Supplement: Supplementary file 1 [file ijms-26-05655-s001.zip › Visual Abstract.jpg]
